# Supplementary material for: Obesity-related T cell dysfunction impairs immunosurveillance and increases cancer risk
Source: Nat Commun. 2024 Apr 2;15:2835. doi: 10.1038/s41467-024-47359-5 (PMC10987624; doi:10.1038/s41467-024-47359-5)
Supplement: Supplementary file 1 — Supplementary Information [file 41467_2024_47359_MOESM1_ESM.pdf]

# Supplementary Information

# Obesity-related T cell dysfunction impairs immunosurveillance and increases cancer risk

**Authors:** Alexander Piening, Emily Ebert, Carter Gottlieb, Niloufar Khojandi, Lindsey M. Kuehm, Stella G. Hoft, Kelly D. Pyles, Kyle S. McCommis, Richard J. DiPaolo, Stephen T. Ferris, Elise Alspach, Ryan M. Teague*

*Corresponding author email: ryan.teague@health.slu.edu

# Supplementary Figure S1

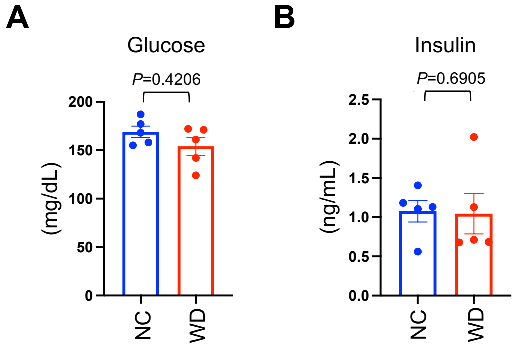


**Supplementary Figure S1:** Mice were placed on either normal chow (NC) or western diet (WD) for 12 weeks. At that time, mice were fasted for 3 hours before serum (A) glucose and (B) insulin levels were measured (NC n=5, WD n=5). For all bar graphs, each point represents an individual mouse with SEM indicated by the error bars. Exact *P* values were calculated by two-sided Mann-Whitney *U* test.

# Supplementary Figure S2

**A**

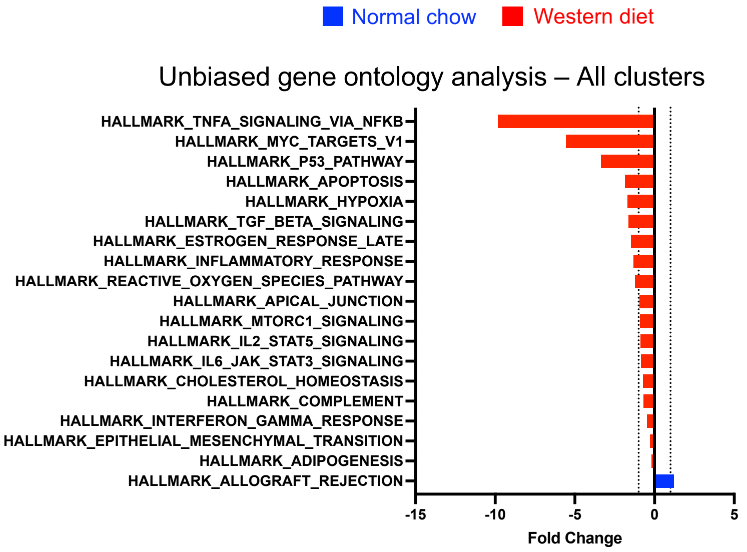


**B**

| Pathway | P-value | Fold change |
|---|---|---|
| HALLMARK_TNFA_SIGNALING_VIA_NFKB | 2.31E-07 | -9.810151 |
| HALLMARK_MYC_TARGETS_V1 | 6.55E-07 | -5.5525126 |
| HALLMARK_P53_PATHWAY | 4.79E-06 | -3.3593437 |
| HALLMARK_APOPTOSIS | 3.09E-05 | -1.8341408 |
| HALLMARK_HYPOXIA | 4.85E-12 | -1.6680639 |
| HALLMARK_TGF_BETA_SIGNALING | 0.00397 | -1.6079465 |
| HALLMARK_ESTROGEN_RESPONSE_LATE | 1.23E-05 | -1.4649321 |
| HALLMARK_INFLAMMATORY_RESPONSE | 7.92E-08 | -1.3183318 |
| HALLMARK_REACTIVE_OXYGEN_SPECIES_PATHWAY | 6.55E-07 | -1.2065217 |
| HALLMARK_APICAL_JUNCTION | 0.00397 | -0.9211269 |
| HALLMARK_MTORC1_SIGNALING | 1.80E-06 | -0.9140131 |
| HALLMARK_IL2_STAT5_SIGNALING | 0.00089 | -0.8830993 |
| HALLMARK_IL6_JAK_STAT3_SIGNALING | 4.79E-06 | -0.8526852 |
| HALLMARK_CHOLESTEROL_HOMEOSTASIS | 0.01568 | -0.7186951 |
| HALLMARK_COMPLEMENT | 4.79E-06 | -0.6949577 |
| HALLMARK_INTERFERON_GAMMA_RESPONSE | 7.49E-05 | -0.4698092 |
| HALLMARK_PEROXISOME | 0.01568 | -0.3370136 |
| HALLMARK_EPITHELIAL_MESENCHYMAL_TRANSITION | 0.01568 | -0.2898063 |
| HALLMARK_ADIPOGENESIS | 0.0004 | -0.181533 |
| HALLMARK_ALLOGRAFT_REJECTION | 2.63E-09 | 1.21519337 |

**C**

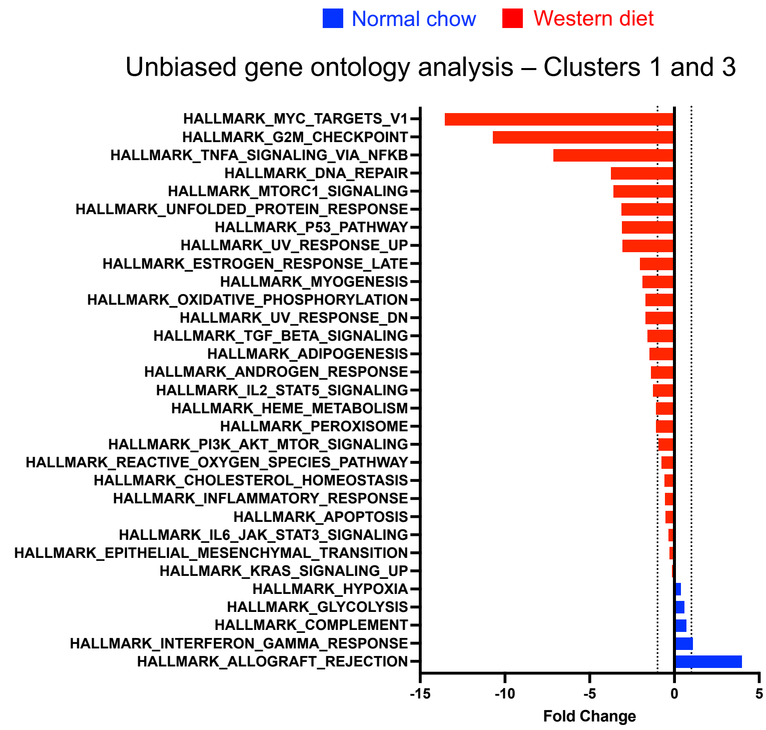


**D**

| Pathway | P-value | Fold change |
|---|---|---|
| HALLMARK_MYC_TARGETS_V1 | 4.79E-06 | -13.538083 |
| HALLMARK_G2M_CHECKPOINT | 4.79E-06 | -10.687153 |
| HALLMARK_TNFA_SIGNALING_VIA_NFKB | 1.92E-16 | -7.1369088 |
| HALLMARK_DNA_REPAIR | 0.0001762 | -3.7500101 |
| HALLMARK_MTORC1_SIGNALING | 8.96E-16 | -3.5771559 |
| HALLMARK_UNFOLDED_PROTEIN_RESPONSE | 0.0004018 | -3.1061193 |
| HALLMARK_P53_PATHWAY | 6.61E-11 | -3.1008447 |
| HALLMARK_UV_RESPONSE_UP | 2.63E-08 | -3.0569757 |
| HALLMARK_ESTROGEN_RESPONSE_LATE | 1.23E-05 | -2.0373894 |
| HALLMARK_MYOGENESIS | 0.0008886 | -1.8774308 |
| HALLMARK_OXIDATIVE_PHOSPHORYLATION | 0.0004018 | -1.699182 |
| HALLMARK_UV_RESPONSE_DN | 0.0004018 | -1.6851626 |
| HALLMARK_TGF_BETA_SIGNALING | 0.0001762 | -1.5908114 |
| HALLMARK_ADIPOGENESIS | 0.0008886 | -1.4762573 |
| HALLMARK_ANDROGEN_RESPONSE | 0.0298094 | -1.3669536 |
| HALLMARK_IL2_STAT5_SIGNALING | 6.61E-11 | -1.2469363 |
| HALLMARK_HEME_METABOLISM | 0.0156844 | -1.083145 |
| HALLMARK_PEROXISOME | 0.0298094 | -1.0695359 |
| HALLMARK_PI3K_AKT_MTOR_SIGNALING | 0.0298094 | -0.9343376 |
| HALLMARK_REACTIVE_OXYGEN_SPECIES_PATHWAY | 0.0004018 | -0.7667961 |
| HALLMARK_CHOLESTEROL_HOMEOSTASIS | 0.0019063 | -0.5780767 |
| HALLMARK_INFLAMMATORY_RESPONSE | 7.49E-05 | -0.5430823 |
| HALLMARK_APOPTOSIS | 6.55E-07 | -0.5262974 |
| HALLMARK_IL6_JAK_STAT3_SIGNALING | 0.0008886 | -0.3359172 |
| HALLMARK_EPITHELIAL_MESENCHYMAL_TRANSITION | 4.79E-06 | -0.29115 |
| HALLMARK_KRAS_SIGNALING_UP | 1.23E-05 | -0.1206735 |
| HALLMARK_HYPOXIA | 7.96E-18 | 0.37638642 |
| HALLMARK_GLYCOLYSIS | 0.0019063 | 0.57180268 |
| HALLMARK_COMPLEMENT | 6.61E-11 | 0.71103851 |
| HALLMARK_INTERFERON_GAMMA_RESPONSE | 1.82E-11 | 1.07935422 |
| HALLMARK_ALLOGRAFT_REJECTION | 4.85E-12 | 3.96311122 |

**Supplementary Figure S2:** Mice were placed on either normal chow (NC) or western diet (WD) for 12 weeks. scRNAseq was performed on $CD8^+$ TIL sorted from day 14 established B16 tumors. (A, B) Unbiased gene ontology was performed on all $CD8^+$ TIL, and significant pathways are shown along with respective *P* values and fold changes. (C, D) Unbiased gene ontology was performed on cytolytic $CD8^+$ TIL clusters 1 and 3, and significant pathways are shown along with respective *P* values and fold changes.

# Supplementary Figure S3

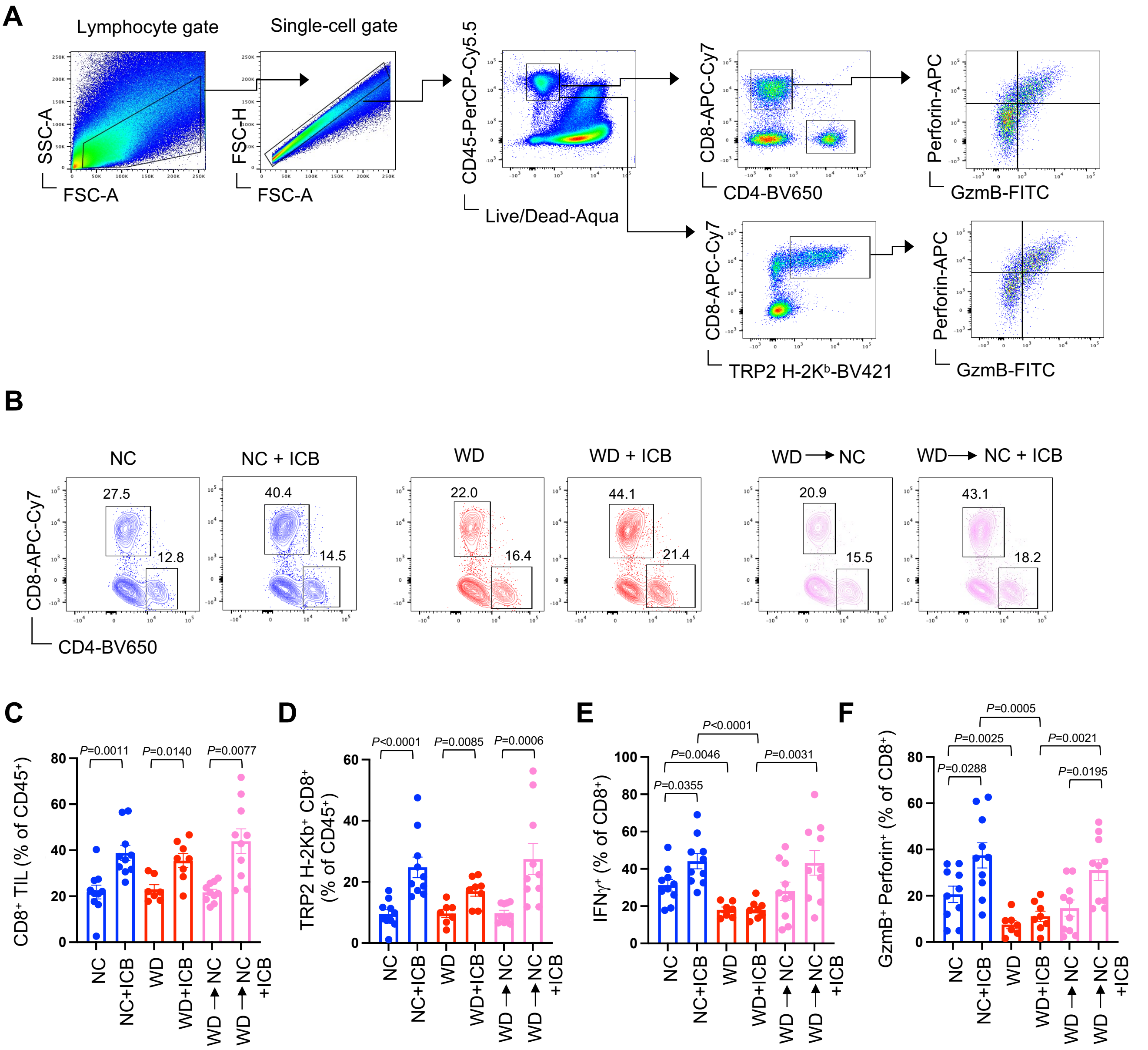


**Supplementary Figure S3:** (A) Gating strategy for ex vivo analysis of CD8$^{+}$ TIL, TRP-2-specific CD8$^{+}$ TIL, and CD4$^{+}$ TIL from B16 melanoma tumors at day 15. (B) Representative FACS plots show TIL that were gated for CD45$^{+}$ expression and display the frequency of CD8$^{+}$ and CD4$^{+}$ T cells from untreated normal chow (NC) and western diet (WD) mice as well as immune checkpoint blockade (ICB) treated cohorts. Graphs display pooled data from 2 independent experiments showing the frequency of (C) total CD8$^{+}$ TIL, (D) TRP-2-specific CD8$^{+}$ TIL, (E) IFNγ$^{+}$ CD8$^{+}$ TIL, and (F) CD8$^{+}$ TIL co-expressing GzmB and Perforin (NC n=10, NC+ICB n=10, WD n=7, WD+ICB n=8, WD→NC n=10, WD→NC+ICB n=10). For all bar graphs, each point represents an individual mouse with SEM indicated by the error bars. Exact *P* values were calculated by two-sided Mann-Whitney *U* test.

# Supplementary Figure S4

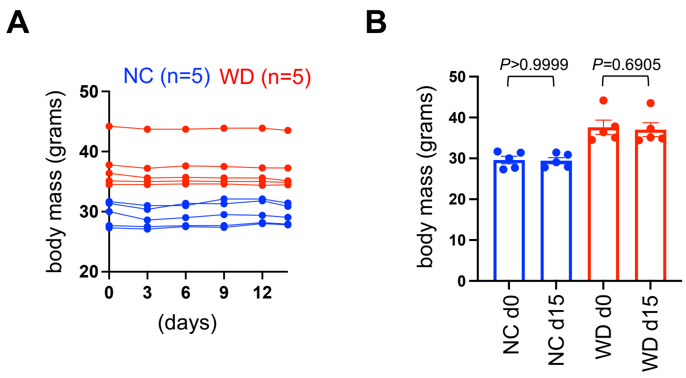


**Supplementary Figure S4:** Mice were placed on either normal chow (NC) or western diet (WD) for 12 weeks. At this time, mice were weighed and then received a subcutaneous injection of B16 tumor cells in the flank (day 0). (A) Mice were weighed every 3 days until takedown on day 15. (B) Day 15 body mass was calculated by subtracting tumor mass from body mass (NC n=5, WD n=5). For all bar graphs, each point represents an individual mouse with SEM indicated by the error bars. Exact *P* values were calculated by two-sided Mann-Whitney *U* test.

# Supplementary Figure S5

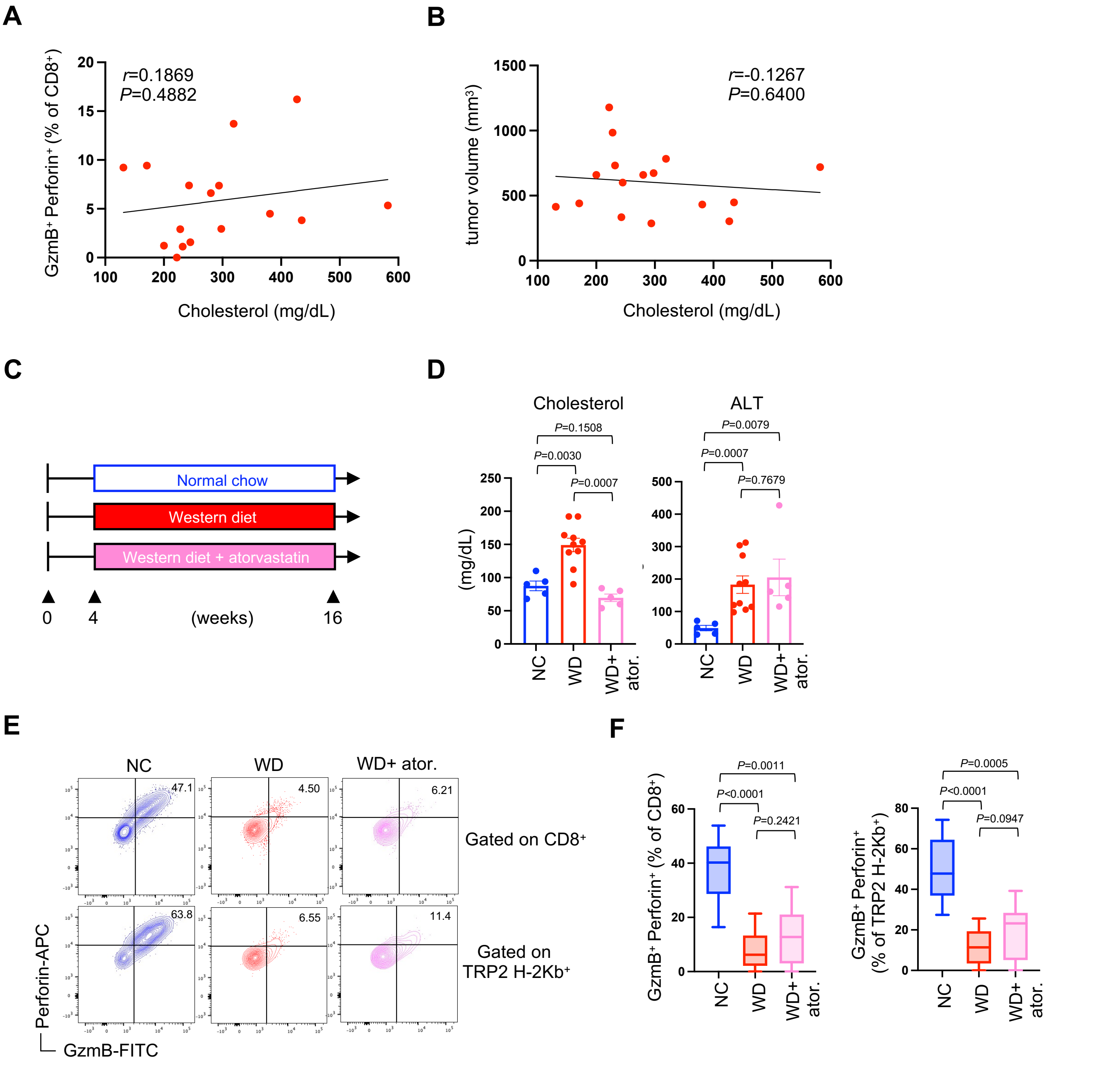


**Supplementary Figure S5:** Linear regression analysis comparing (A) the frequency of $CD8^+$ TIL co-expressing GzmB and Perforin, or (B) tumor volumes to mouse serum cholesterol in western diet (WD) mice (pooled data from 3 independent experiments, n=16). (C) Mice were placed on either normal chow (NC), WD, or WD containing atorvastatin for 12 weeks. (D) Serum cholesterol and alanine aminotransferase (ALT) levels were measured (NC n=5, WD n=10, WD+ator. n=5). At this time, mice received a subcutaneous injection of B16 tumor cells in the flank. (E) Tumors were harvested at day 16 and representative FACS plots show intracellular expression of GzmB and Perforin from total $CD8^+$ TIL and TRP-2-specific $CD8^+$ TIL. (F) Pooled data from 2 independent experiments show the percent of total $CD8^+$ TIL and TRP-2-specific $CD8^+$ TIL co-expressing GzmB and Perforin (NC n=12, WD n=19, WD+ator. n=8). (A, B) Linear regression analysis was performed by calculating the Pearson correlation value (*r)* and corresponding *P* value. Each point represents an individual mouse. (D, F) For all bar graphs, each point represents an individual mouse with SEM indicated by the error bars. All box-and-whisker plots: The box indicates the $25^{th}$ and $75^{th}$ percentile, the line indicates the data median, and the whiskers indicate the minimum and maximum of all individual values. All *n*'s represent an individual mouse. Exact *P* values were calculated by two-sided Mann-Whitney *U* test.

# Supplementary Figure S6

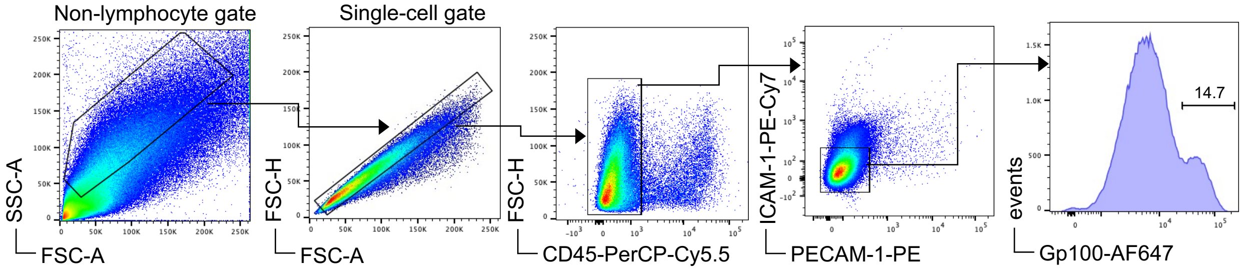


**Supplementary Figure S6:** Gating strategy for ex vivo Gp100+ tumor cells. Tumor cells were distinguished from leukocytes, stromal endothelial cells, and fibroblasts via lack of CD45, ICAM-1, and PECAM-1 surface expression.

# Supplemental Figure S7

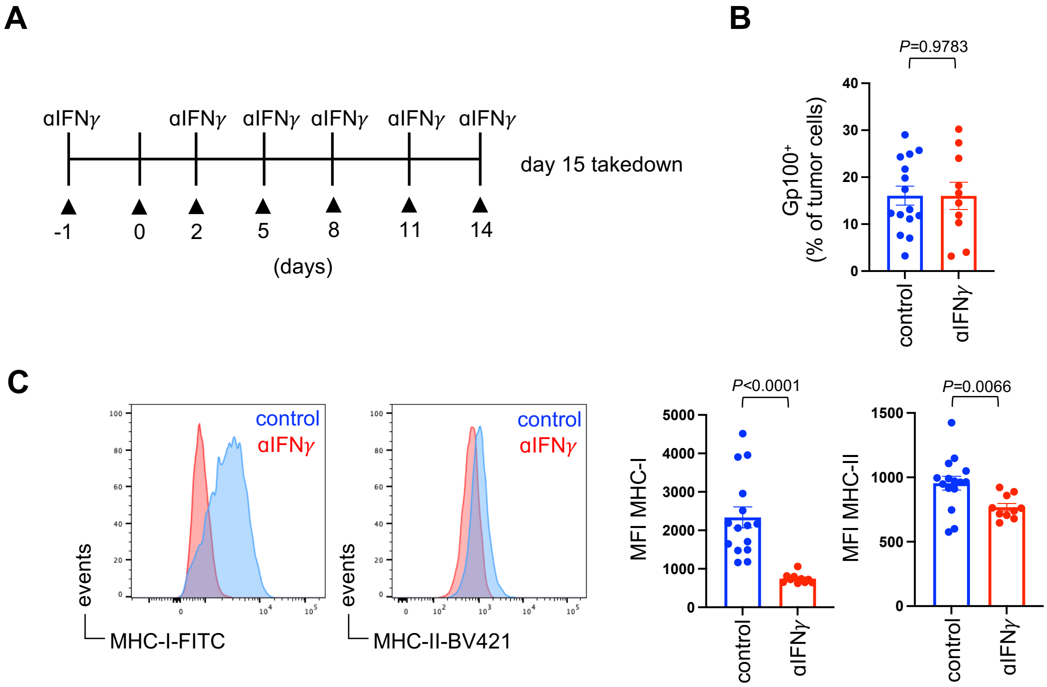


**Supplementary Figure S7:** (A) B6 mice were treated with either αIFNγ antibody or vehicle control via intraperitoneal injection on days -1, 2, 5, 8, 11, and 14 relative to subcutaneous injection of B16 tumor cells in the flank on day 0. (B) Tumors were harvested on day 15 and the frequency of Gp100$^{+}$ tumor cells was measured. (C) Representative FACS plots show MHC-I and MHC-II expression on Gp100$^{+}$ tumor cells, and pooled median fluorescence intensity (MFI) data from 2 independent experiments is graphed (control n=10, αIFNγ n=10). For all bar graphs, each point represents an individual mouse with SEM indicated by the error bars. Exact *P* values were calculated by two-sided Mann-Whitney *U* test.

# Supplementary Figure S8

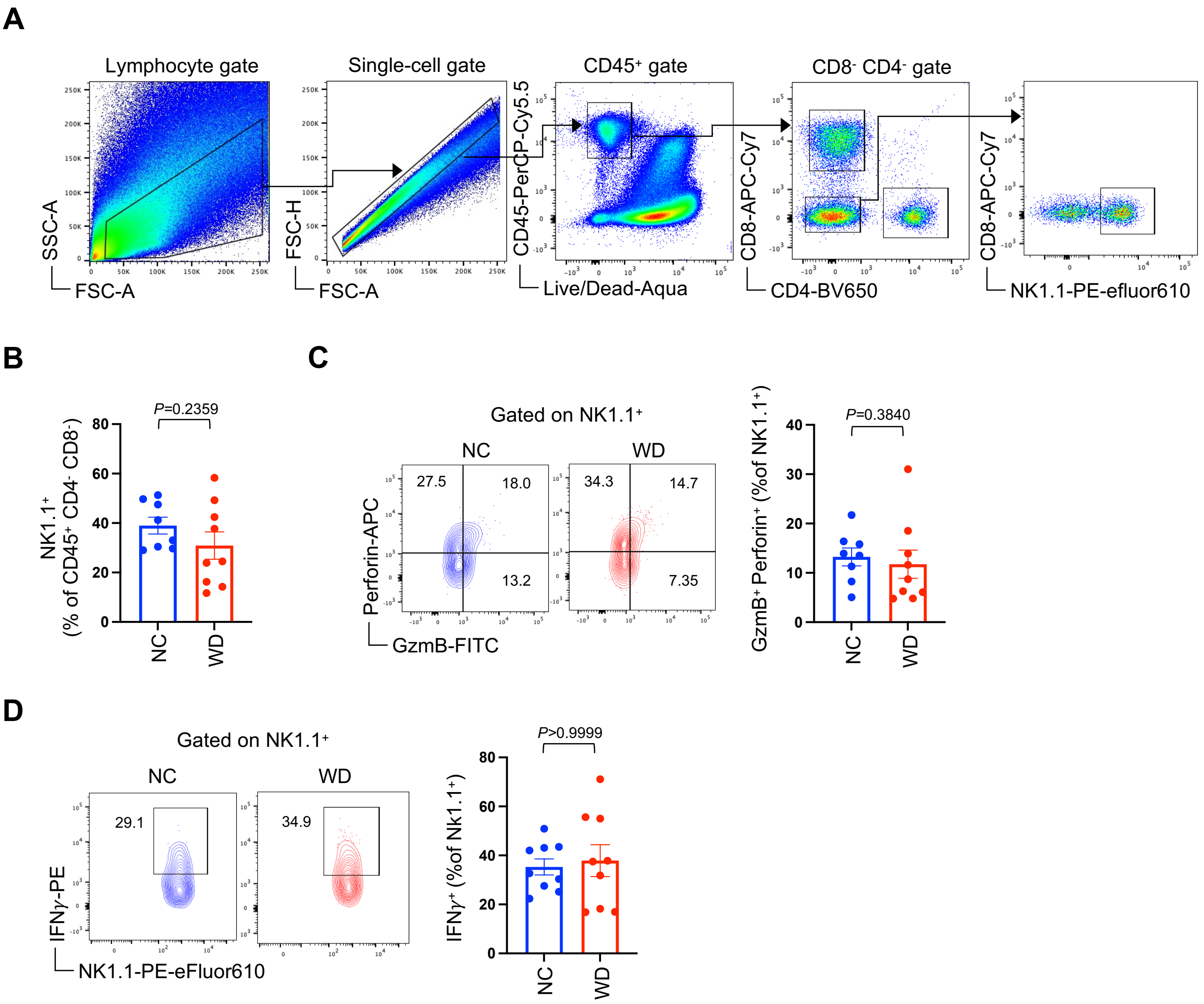


**Supplementary Figure S8:** B16 tumors were harvested from the indicated mice at day 15. (A) Gating strategy for NK1.1$^+$ natural killer (NK) cells from ex vivo B16 melanoma tumors. (B) Graphs display pooled data from 2 independent experiments showing the frequency of NK cells from normal chow (NC) and western diet (WD) tumors. Representative FACS plots and graphs show (C) GzmB and Perforin co-expression and (D) IFN$\gamma$ expression by NK cells (NC n=10, WD n=10). For all bar graphs, each point represents an individual mouse with SEM indicated by the error bars. Exact *P* values were calculated by two-sided Mann-Whitney *U* test.

# Supplementary Figure S9

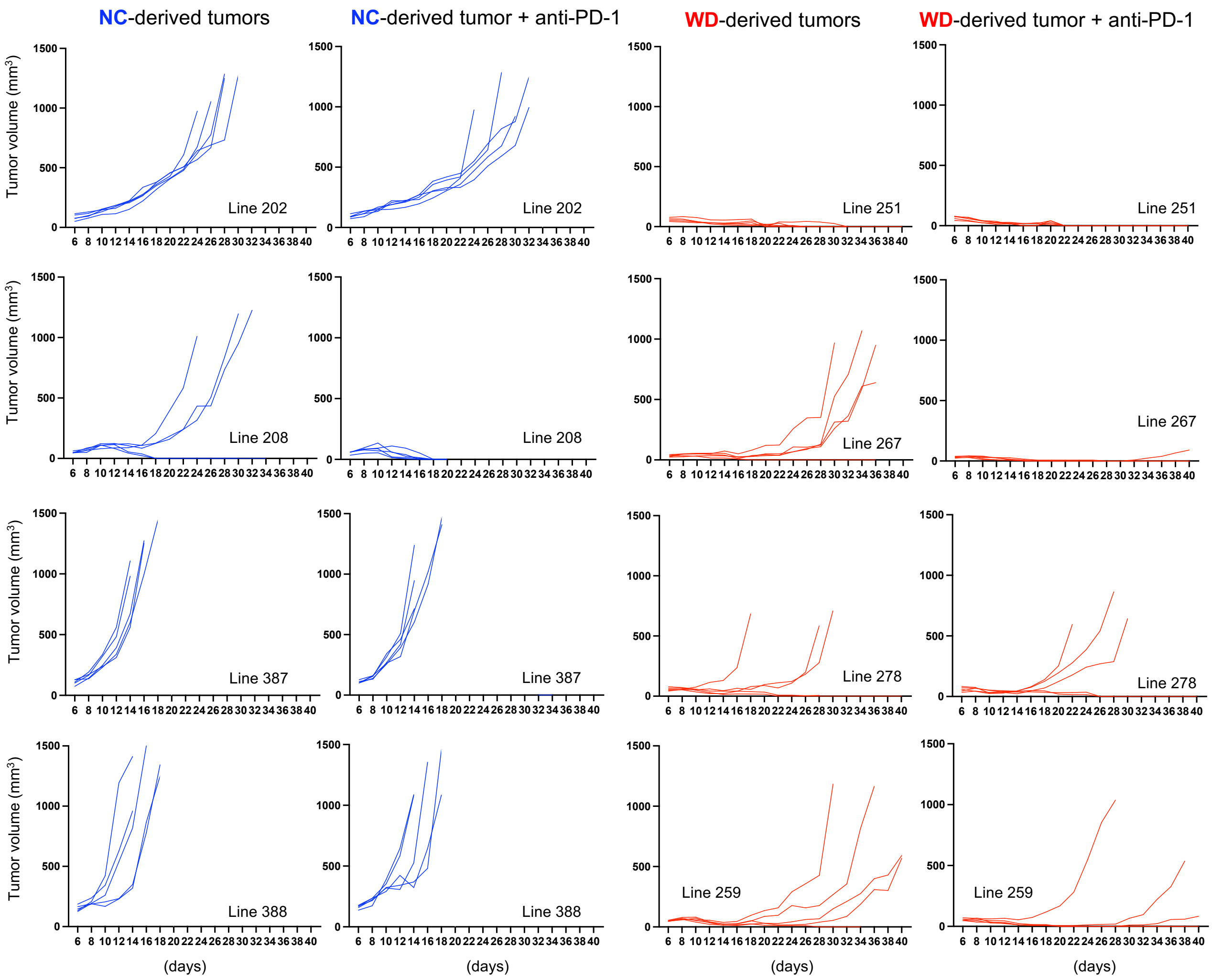


**Supplementary Figure S9:** Individual growth curves for the 4 normal chow (NC)-derived and 4 western diet (WD)-derived sarcoma lines following transplant into 5 secondary B6 recipients left untreated (PBS) and 5 treated with anti-PD-1 on days 7 and 14 (all groups n=5). Each graph represents a unique MCA-induced sarcoma cell line derived from an individual mouse in Figure 5B.

# Supplementary Figure S10

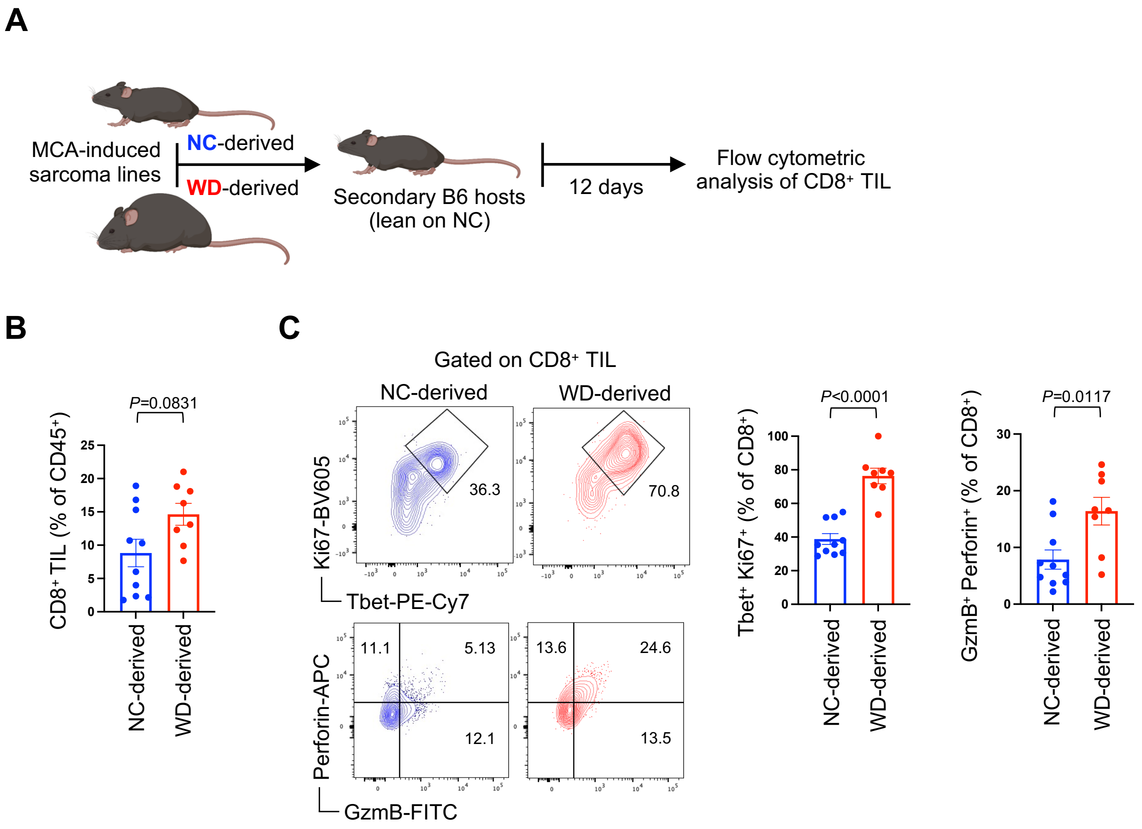


**Supplementary Figure S10:** (A) MCA-induced sarcoma cell lines were generated from mice on normal chow (NC) and mice on western diet (WD) for reimplantation into secondary lean recipients. Two NC-derived (Lines 202 and 387) and two WD-derived (Lines 267 and 278) lines were injected subcutaneously into the flank of 5 secondary recipients each, and tumors were harvested for flow cytometric analysis of $CD8^+$ TIL on day 12, and (B) the frequency of $CD8^+$ TIL was assessed. (C) Representative FACS plots show co-expression of GzmB and Perforin as well as Tbet and Ki67 from $CD8^+$ TIL, and pooled data is graphed (NC-derived n=10, WD-derived n=8). For all bar graphs, each point represents an individual mouse with SEM indicated by the error bars. Exact $P$ values were calculated by two-sided Mann-Whitney $U$ test. (A) Created using BioRender.com.
