# Supplementary material for: Obesity-related T cell dysfunction impairs immunosurveillance and increases cancer risk
Source: Nat Commun. 2024 Apr 2;15:2835. doi: 10.1038/s41467-024-47359-5 (PMC10987624; doi:10.1038/s41467-024-47359-5)
Supplement: Supplementary file 3 — Reporting Summary [file 41467_2024_47359_MOESM3_ESM.pdf]

nature portfolio

Corresponding author(s): Ryan M. Teague

Last updated by author(s): 3/15/2024

Reporting Summary

Nature Portfolio wishes to improve the reproducibility of the work that we publish. This form provides structure for consistency and transparency in reporting. For further information on Nature Portfolio policies, see our Editorial Policies and the Editorial Policy Checklist.

Statistics

For all statistical analyses, confirm that the following items are present in the figure legend, table legend, main text, or Methods section.

n/a	Confirmed	
☐	☒	The exact sample size (*n*) for each experimental group/condition, given as a discrete number and unit of measurement
☐	☒	A statement on whether measurements were taken from distinct samples or whether the same sample was measured repeatedly
☐	☒	The statistical test(s) used AND whether they are one- or two-sided<br>*Only common tests should be described solely by name; describe more complex techniques in the Methods section.*
☒	☐	A description of all covariates tested
☒	☐	A description of any assumptions or corrections, such as tests of normality and adjustment for multiple comparisons
☐	☒	A full description of the statistical parameters including central tendency (e.g. means) or other basic estimates (e.g. regression coefficient) AND variation (e.g. standard deviation) or associated estimates of uncertainty (e.g. confidence intervals)
☐	☒	For null hypothesis testing, the test statistic (e.g. *F*, *t*, *r*) with confidence intervals, effect sizes, degrees of freedom and *P* value noted<br>*Give P values as exact values whenever suitable.*
☒	☐	For Bayesian analysis, information on the choice of priors and Markov chain Monte Carlo settings
☒	☐	For hierarchical and complex designs, identification of the appropriate level for tests and full reporting of outcomes
☒	☐	Estimates of effect sizes (e.g. Cohen's *d*, Pearson's *r*), indicating how they were calculated

Our web collection on statistics for biologists contains articles on many of the points above.

Software and code

Policy information about availability of computer code

Data collection	Illumina sequencing and quality control of libraries were performed on the NovaSeq6000 platform at the Washington University in St. Louis Genome Technology Access Center (GTAC). No custom software or code were used in the collection of data in this study.
Data analysis	Raw data were processed through the CellRanger 6.0.2 pipeline (10x Genomics), clustering and differential expression analysis were conducted using R v2023.09.1+494 and open source software package Seurat v4.3.0, and visualization was conducted using ggplot2 v3.4.3. Unbiased Hallmark pathway analysis was conducted using the msigdbr v7.5.1 and SCPA v1.5.4 packages.

For manuscripts utilizing custom algorithms or software that are central to the research but not yet described in published literature, software must be made available to editors and reviewers. We strongly encourage code deposition in a community repository (e.g. GitHub). See the Nature Portfolio guidelines for submitting code & software for further information.

Data

Policy information about availability of data

All manuscripts must include a data availability statement. This statement should provide the following information, where applicable:

- Accession codes, unique identifiers, or web links for publicly available datasets
- A description of any restrictions on data availability
- For clinical datasets or third party data, please ensure that the statement adheres to our policy

Single-cell RNA sequencing data has been uploaded to Gene Expression Omnibus (GEO) for public access with the accession number GSE245657 (https://www.ncbi.nlm.nih.gov/geo/query/acc.cgi?acc=GSE245657).

Research involving human participants, their data, or biological material

Policy information about studies with human participants or human data. See also policy information about sex, gender (identity/presentation), and sexual orientation and race, ethnicity and racism.

Reporting on sex and gender	No human participants or human data were involved in these studies.
Reporting on race, ethnicity, or other socially relevant groupings	No human participants or human data were involved in these studies.
Population characteristics	No human participants or human data were involved in these studies.
Recruitment	No human participants or human data were involved in these studies.
Ethics oversight	No human participants or human data were involved in these studies.

Note that full information on the approval of the study protocol must also be provided in the manuscript.

Field-specific reporting

Please select the one below that is the best fit for your research. If you are not sure, read the appropriate sections before making your selection.

☒ Life sciences ☐ Behavioural & social sciences ☐ Ecological, evolutionary & environmental sciences

For a reference copy of the document with all sections, see nature.com/documents/nr-reporting-summary-flat.pdf

Life sciences study design

All studies must disclose on these points even when the disclosure is negative.

Sample size	No sample size calculations were performed. The studies aimed to have as many replicates as possible, with the goal of each experiment to have a minimum of 5 biological replicates per group to ensure reproducibility of our findings.
Data exclusions	No data was excluded.
Replication	Experimental replication information is annotated in Figure legends, with the majority of experiments being replicated a minimum of 2 times.
Randomization	Mice were randomly assigned to either control or experimental groups when received from Jackson Labs.
Blinding	Blinding was not possible in these studies as mouse cohorts belonged to discrete dietary groups, easily identifying them to a specific experimental arm.

Reporting for specific materials, systems and methods

We require information from authors about some types of materials, experimental systems and methods used in many studies. Here, indicate whether each material, system or method listed is relevant to your study. If you are not sure if a list item applies to your research, read the appropriate section before selecting a response.

Materials & experimental systems

n/a	Involved in the study
☐	☒ Antibodies
☐	☒ Eukaryotic cell lines
☒	☐ Palaeontology and archaeology
☐	☒ Animals and other organisms
☒	☐ Clinical data
☒	☐ Dual use research of concern
☒	☐ Plants

Methods

n/a	Involved in the study
☒	☐ ChIP-seq
☐	☒ Flow cytometry
☒	☐ MRI-based neuroimaging

Antibodies

Antibodies used	Fluorochrome-conjugated antibodies were purchased from Biolegend (anti-CD45, cat. #157612, clone QA17A26, host mouse, reactivity mouse, verified for flow cytometry, used for flow cytometry, dilution 1:200; anti-CD8a, cat. #100714, clone 53-6.7, host rat, reactivity mouse, verified for flow cytometry, used for flow cytometry, dilution 1:200; anti-CD4, cat. #100546, clone RM4-5, host rat, reactivity mouse, verified for flow cytometry, used for flow cytometry, dilution 1:200; anti-H-2Kb/H-2Db, cat. #114606, clone 28-8-6, host mouse, reactivity mouse, verified for flow cytometry, used for flow cytometry, dilution 1:200; anti-PD-1, cat. #135231, clone 29F.1A12, host rat, reactivity mouse, verified for flow cytometry, used for flow cytometry, dilution 1:200; anti-TIM3, cat. #119721, clone RMT3-23, host rat, reactivity mouse, verified for flow cytometry, used for flow cytometry, dilution 1:200; anti-LAG-3, cat. #125208, clone C9B7W, host rat, reactivity mouse, verified for flow cytometry, used for flow cytometry, dilution 1:200; anti-PECAM-1, cat. #102507, clone MEC13.3, host rat, reactivity mouse, verified for flow cytometry, used for flow cytometry, dilution 1:200; anti-ICAM-1, cat. #116121, clone YN/1.7.4, host rat, reactivity mouse, verified for flow cytometry, used for flow cytometry, dilution 1:200; Granzyme B, cat. #515403, clone GB11, host mouse, reactivity human/mouse, verified for intracellular flow cytometry, used for intracellular flow cytometry, dilution 1:100; Perforin, cat. #154304, clone S16009A, host rat, reactivity mouse, verified for intracellular flow cytometry, used for intracellular flow cytometry, dilution 1:100; anti-IFNg, cat. #505808, clone XMG1.2, host rat, reactivity mouse, verified for intracellular flow cytometry, used for intracellular flow cytometry, dilution 1:100), Thermo Fisher Scientific (anti-NK1.1, cat. #61-5941-82, clone PK136, host mouse, reactivity mouse, verified for flow cytometry, used for flow cytometry, dilution 1:200), BD Biosciences (anti-MHC-II I-ab, cat. #562928, clone AF6-120.1, host mouse, reactivity mouse, verified for flow cytometry, used for flow cytometry,dilution 1:200), Invitrogen (anti-IFNgR, cat. #12-1191-82, clone 2E2, host Armenian hamster, reactivity mouse, verified for flow cytometry, used for flow cytometry, dilution 1:200), and Abcam (Gp100, cat. #246730, clone EP4863(2), host rabbit, reactivity mouse, verified for intracellular flow cytometry, used for intracellular flow cytometry, dilution 1:100). All flow cytometry verified antibodies were used at a 1:200 dilution and all intracellular flow cytometry verified antibodies were used at a 1:100 dilution. In vivo antibodies anti-PD-1 (clone RMP1-14, cat. #BE0146) and anti-CTLA-4 (clone 9D9, cat. #BE0164) were obtained from Bio X Cell and were used at a concentration of 5mg/kg. In vivo neutralizing antibody anti-IFNg (clone XMG1.2, cat. #BE0055) was obtained from Bio X Cell and 200ug was administered per injection. In vivo depleting antibody anti-Thy1.2 (clone 30H12, cat. #BE0164) was obtained from Bio X Cell and 200ug was administered per injection.
Validation	Antibodies used for flow cytometry and intracellular flow cytometry were validated by the respective suppliers as indicated by the supplier websites. Technical datasheets are available for each antibody and can be accessed on the supplier websites using the provided catalogue number as a reference. In vivo antibodies were validated by the suppliers as indicated by the supplier websites, and anti-Thy1.2 in vivo depleting antibodies were validated via flow cytometry using anti-CD8a (Biolegend, cat. #100714, clone 53-6.7).

Eukaryotic cell lines

Policy information about cell lines and Sex and Gender in Research

Cell line source(s)	The B16-F0 (B16 hereafter) and YUMM melanoma tumor cell lines were purchased from the American Tissue Culture Collection (ATCC) (cat. #CRL-6322, cat. #CRL-3362). Primary sarcoma tumor cell lines were generated from injection of male mice with 3'-methylcholanthrene.
Authentication	The B16 cell line was authenticated by LabCorp Genetica Cell Line Testing (Burlington NC, USA) via short tandem repeat (STR) profiling. The YUMM melanoma tumor cell line underwent no authentication after purchase.
Mycoplasma contamination	The B16 cell line was confirmed negative for Mycoplasma on February 2, 2020 by LabCorp Genetica Cell Line Testing (Burlington NC, USA). Cell lines other than the B16 cell line were not confirmed free of mycoplasma contamination.
Commonly misidentified lines (See ICLAC register)	No commonly misidentified cell lines were used in these studies.

Animals and other research organisms

Policy information about studies involving animals; ARRIVE guidelines recommended for reporting animal research, and Sex and Gender in Research

Laboratory animals	C57BL/6 (Strain No. 000664), OT-II (Strain No. 004194) and Rag2-/- (Strain No. 033526) mice were purchased from the Jackson Laboratory. Mice were age-matched and sex-matched and between 2 and 10 months of age when used for experiments. Housing light cycle (12 hours starting 6 AM CST) and dark cycle (12 hours starting 6 PM CST), ambient temperature of 72 degrees Fahrenheit.

Wild animals	No studies involved wild animals.
Reporting on sex	Figure 1 utilized data from male mice, Supplemental Figure S1 utilized data from male mice, Supplemental Figure S2 utilized data from male mice, Figure 2 and Supplemental Figure S3 utilized data from equal numbers of male and female mice, Supplemental Figure S4 utilized data from male mice, Figure 3 utilized data from male mice, Supplemental Figure S5 utilized data from male mice, Figure 4 utilized data from male mice, Supplemental Figures S7 and S8 utilized data from male mice, Figure 5 utilized data from male mice, Figure 6 utilized data from male mice, and Supplemental Figure S9 and S10 utilized data from male mice,. Preliminary experiments done in 4-times replicate confirmed a similar CD8+ tumor-infiltrating lymphocyte defect phenotype between male and female mice, after which male mice were predominantly used in experiments.
Field-collected samples	No studies involved field collected samples.
Ethics oversight	All mice were housed under pathogen-free conditions in the Saint Louis University School of Medicine Department of Comparative Medicine and used in accordance with animal use protocols approved by the Institutional Animal Care and Use Committee.

Note that full information on the approval of the study protocol must also be provided in the manuscript.

Plants

Seed stocks	No plants were involved in these studies.
Novel plant genotypes	No plants were involved in these studies.
Authentication	No plants were involved in these studies.

Flow Cytometry

Plots

Confirm that:

☒ The axis labels state the marker and fluorochrome used (e.g. CD4-FITC).

☒ The axis scales are clearly visible. Include numbers along axes only for bottom left plot of group (a 'group' is an analysis of identical markers).

☒ All plots are contour plots with outliers or pseudocolor plots.

☒ A numerical value for number of cells or percentage (with statistics) is provided.

Methodology

Sample preparation	For analysis of tumor cells and tumor-infiltrating lymphocytes via flow cytometry, mice were sacrificed, and tumors were excised, mechanically disrupted with a sterile 3-mL syringe plunger and filtered through a 40-um cell strainer. Isolation steps were performed in cold PBS. Intracellular staining of cytoplasmic- and nuclear-associated proteins was performed using the eBioscience cellular permeabilization kit (cat. #00-5523-00) per the manufacturer's instructions. Briefly, cells were processed and stained ex vivo with live/dead and cell surface markers described above. Cells were then fixed, permeabilized, and stained with antibodies specific for the intracellular proteins Granzyme B (Biolegend; cat. #515403, clone GB11, host mouse, reactivity human/mouse, verified for intracellular flow cytometry, used for intracellular flow cytometry, dilution 1:100), Perforin (Biolegend; cat. #154304, clone S16009A, host rat, reactivity rat, verified for intracellular flow cytometry, used for intracellular flow cytometry, dilution 1:100), or Gp100 (Abcam; cat. #246730, clone EP4863(2), host rabbit, reactivity mouse, verified for intracellular flow cytometry, used for intracellular flow cytometry, dilution 1:100). Intracellular cytokine staining was performed using the Cytofix/Cytoperm plus kit (BD Biosciences) per the manufacturer's instructions. Briefly, cells were incubated ex vivo with 10 ng/mL PMA (Sigma-Aldrich; cat. #P8139) and 0.3 ug Ionomycin (Sigma-Aldrich; cat. #I0634) for 4 hours in the presence of GolgiPlug (BD Biosciences; 51-2301KZ). Cells were first stained with live-dead and cell surface markers as described above, then fixed, permeabilized, and stained with anti-IFNg (Biolegend; cat. #505808, clone XMG1.2, host rat, reactivity mouse, verified for intracellular flow cytometry, used for intracellular flow cytometry, dilution 1:100). All intracellular flow cytometry antibodies were used at a 1:100 dilution.
Instrument	Flow-cytometric analysis was performed on LSRFortessa X-20 3L Cell Analyzer (BD Biosciences) in the Saint Louis University Flow Cytometry Core Facility.
Software	Data was analyzed using using FlowJo v.10 software (Tree Star Inc.).
Cell population abundance	Cell sorting was performed to at least 99% purity based on CD8 staining using a BD FACSAria III (BD Biosciences).
Gating strategy	Gating for tumor-infiltrating CD8+ and CD4+ T cells: The starting cell population in the preliminary FSC/SSC gates was whole

Gating strategy

tumor, a gate was drawn for low SSC and medium FSC cells, a single-cell gate was drawn, a live, CD45+ gate was drawn, and then gating was done for CD8+ cells, CD4+ cells, and TRP2 H-2Kb+ cells (See Supplemental Figure S3). Gating for tumor cells: The starting cell population in the preliminary FSC/SSC gates was whole tumor, a single-cell gate was drawn, a CD45- gate was drawn, and a ICAM-1- PECAM-1- gate was drawn to identify tumor cells (See Supplemental Figure S6). Gating for natural killer cells: The starting cell population in the preliminary FSC/SSC gates was whole tumor, a gate was drawn for low SSC and medium FSC cells, a single-cell gate was drawn, a live, CD45+ gate was drawn, a CD4- CD8- gate was drawn, and then gating was done for NK1.1+ cells (See Supplemental Figure S8).

☒ Tick this box to confirm that a figure exemplifying the gating strategy is provided in the Supplementary Information.
